# A comprehensive evaluation of the effect of key parameters on the performance of DTPA chelating agent in modifying sandstone surface charge

**DOI:** 10.1016/j.heliyon.2023.e21990

**Published:** 2023-11-02

**Authors:** Mahsa Parhizgar Keradeh, Seyyed Alireza Tabatabaei-Nezhad

**Affiliations:** Faculty of Petroleum and Natural Gas Engineering, Sahand University of Technology, Sahand, Tabriz, PO.Box: 51335-1996, Iran

**Keywords:** DTPA chelating agent, EOR, Wettability alteration, Zeta potential, Sand pack flooding

## Abstract

Despite the positive aspects of low salinity water (LSW), this technique is relatively expensive and unavailable in some countries. Furthermore, potential problems associated with LSW such as scale precipitation in carbonate reservoirs and fine migration in sandstone reservoirs raise concerns. Chelating agents have the ability to chelate metal ions from solution, effectively reducing the salinity of seawater (SW) and mimicking the behavior of LSW. However, they mitigate the challenges associated with LSW injection. This study focuses on how the Diethylenetriaminepentaacetic acid (DTPA) chelating agent performs in modifying rock surface charge. The impact of concentration, brine salinity, potential determining ions (PDIs), oil presence, Fe^3+^ ions, and solution pH on the effectiveness of DTPA in altering rock surface charge was evaluated. Furthermore, wettability alteration and sand pack flooding tests were conducted to study the effect of DTPA on rock wettability and oil recovery. Results of wettability alteration, zeta potential, sand pack flooding experiments and ion concentration analysis are reported in this paper. The results showed that reducing salinity, increasing DTPA concentration, and raising solution pH changed rock wettability from oil-wetness towards water-wetness. The presence or absence of PDIs in the solution did not affect the performance of DTPA. However, by tripling the concentration of these ions in the solution, the performance of DTPA in changing rock surface charge was impaired. Based on the wettability alteration and zeta potential experiments, 5 wt% DTPA was determined as the optimum concentration. Subsequent flooding experiments revealed that injecting 5 wt% DTPA chelating agents into the sandstone sand pack after SW injection increased oil recovery from 48 % to 68.3 %. The analysis of ion concentrations also revealed a significant increase in the amount of calcium ions during the DTPA flooding, indicating the chelation of metal ions from both rock and solution and improving the wettability conditions.

## 1 Introduction

Despite the development of new enhanced oil recovery (EOR) techniques, waterflooding is the most widely used method for EOR due to its low cost. However, the mixing of seawater (SW) with formation water (FW) can lead to the precipitation of scales such as barite (BaSO_4_), celestine (SrSO_4_), and anhydrite (CaSO_4_) inside the reservoir, flow lines, and surface equipment [1,2]. The injection of low salinity water (LSW) has been shown to reduce capillary pressure, oil/water interfacial tension, and alter rock wettability to more water wetness, leading to changes in relative permeability [3]. However, it is important to note that injecting LSW can also potentially cause reservoir damage. In carbonate reservoirs, damage may result from precipitation, while in sandstone reservoirs, it can be due to fine migration and blockage of pore throats [2,4,5]. Mahmoud (2014) demonstrated that injecting SW into sandstone formations can result in the precipitation of calcium sulfate (CaSO_4_), leading to a reduction in permeability [4]. These problems make us look for an alternative to SW and LSW.

Chelating agents have been widely used in upstream and downstream industries. These agents are employed in various applications such as EOR, well stimulation, and scale removal from oil and gas reservoirs [[6], [7], [8], [9], [10], [11], [12]]. In the oil and gas industry, the term “chelating agents” specifically refers to a sub-group of amino carboxylic acids. Mixing chelating agents in undiluted SW has been suggested as an alternative to LSW flooding. This approach involves chelation and capture of metal ions from SW, resulting in a reduction of SW salinity without the need for any dilution processes. They are not absorbed on the rock surface and do not damage the pores and also prevent precipitation by chelating different metal ions from rock and fluid [7,13,14]. As a result, using chelating agents is an effective way to increase oil recovery and reduce the cost of EOR projects. Chelating agents were first used by Morgan and Drew in the 1920s [15]. The term “chelate” originates from the Greek word "lobster's claw” as chelating agents act like claws, grabbing metal ions from the solution and preventing them from reacting with other compounds [16].

**Fig. 1:**
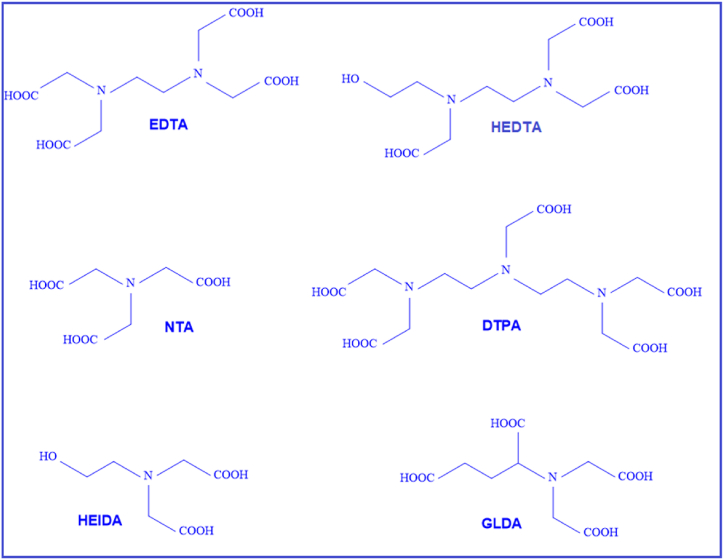
Chelating agents used in the oil and gas industry [22].

Fred and Fogler (1997) were the first to use chelating agents in the oil field. They used EDTA chelating agent to stimulate the sandstone reservoir [17]. Attia et al. (2014) also reported the novel use of chelating agents in EOR applications, suggesting their potential as an alternative to LSW [18]. Chelating agents work by separating metal ions from both SW and FW, which in turn forces the rock to compensate for this ion loss by releasing certain ions, such as ${\text{Ca}}^{2+}$ from its surface. This process leads to rock dissolution and subsequent release of ions from the rock surface, resulting in a change in rock wettability to become water-wet and facilitating the release of oil from the rock surface. Notably, rock dissolution induced by chelating agents is recognized as one of the main mechanisms for EOR [19,20]. Fig. 1 shows some commonly used chelating agents in the oil and gas industry, while Table 1 presents the stability constants for various chelating agents. The stability constant means the dependence of the chelating agent on the metal ion and describes the stability of the bond formed between the chelate and the metal ion. A higher stability constant indicates a more stable chelation product [21]. For instance, in the case of diethylenetriaminepentaacetic acid (DTPA) chelating agent, the stability constant with ${\text{Fe}}^{2+}$ is higher than that with ${\text{Ca}}^{2+}$, indicating that DTPA first separates ${\text{Fe}}^{2+}$ from the rock and solution, and then captures and chelates ${\text{Ca}}^{2+}$ ions. The stability constant is calculated using equations (1), (2).

(1) $$A^{n-}+M^{m+}↔{\text{MA}}^{m-n}$$

(2) $$K_{f}=\log\left(\frac{\left[{\text{MA}}^{m-n}\right]}{\left[A^{n-}\right]\left[M^{m+}\right]}\right)$$

where, A represents the chelating agent, M represents the metal ion, K_f_ is the stability constant (dimensionless), $\left[{\text{MA}}^{m-n}\right]$ is the concentration of the complex, $\left[A^{n-}\right]$ refers to the concentration of the chelating agent and $\left[M^{m+}\right]$ refers the concentration of the metal ion.

**Table 1:** Stability constants of DTPA, EDTA and HEDTA chelating agents with different ions [[23], [24], [25], [26]].

Chelating agent	${\text{Ca}}^{2+}$	${\text{Mg}}^{2+}$	${\text{Fe}}^{2+}$	${\text{Fe}}^{3+}$	${\text{Ba}}^{2+}$	${\text{Sr}}^{2+}$	${\text{Al}}^{3+}$	${\text{Mn}}^{2+}$	${\text{Zn}}^{2+}$	${\text{Cu}}^{2+}$	${\text{Ni}}^{2+}$	${\text{Zr}}^{4+}$
DTPA	10.9	9.3	16.5	28	8.6	9.7	18.4	15.6	18.75	21	20.32	36.9
EDTA	10.7	8.7	14.3	25.7	7.7	8.6	16.1	13.5	17.5	19.7	18.62	29.9
HEDTA	8.4	7	12.2	19.8	6.2	6.92	15.6	10.9	14.7	18.3	17	–

The measurement of contact angle and zeta potential is used to determine wettability alteration and rock surface charge. The stability of the water film, a critical factor affecting rock wettability, is determined by the electrical charge at the rock/brine and oil/brine interfaces [27,28]. Zeta potential measurement is crucial as it provides insight into the electric potential and surface charge. Factors such as pH and ionic strength can significantly impact the zeta potential. For instance, at pH values below 5.5, the zeta potential decreases with increasing salinity, while at pH values above 6, it increases with increasing salinity [29]. Nasralla et al. (2013) conducted experiments to measure the contact angle and zeta potential between oil and sandstone samples. Their findings indicated that reducing the salinity of system led to a decrease in the contact angle between the rock and oil, along with a more negative zeta potential. They reported that using deionized water (DIW) changed the contact angle from 72° to 31° and decreased the zeta potential value from −3 mV to −22 mV [30]. Attia et al. (2014) conducted zeta potential measurements on sandstone samples. Their findings revealed that using the EDTA chelating agent resulted in more negative zeta potential values compared to SW and LSW, leading to a shift in rock wettability towards water-wetness. The researchers attributed this change in wettability to the dissolution of rock and chelation of various ions [18]. Mahmoud and Abdelgawad (2015) mixed 5 wt% EDTA chelating agent with chlorite mineral and observed that due to the chelation of metal ions from chlorite mineral, the zeta potential changed from positive to negative value [19]. Alarifi et al. (2018) performed zeta potential measurements for DTPA chelating agents and water with different salinities. Their results showed that DTPA adsorbed ions such as ${\text{Fe}}^{3+}$ from sandstone rock surfaces and changed the wettability to more water-wet. Also, they reported the same zeta potential value for 5 wt% DTPA/SW and LSW, which means that DTPA eliminates the effects of salt concentration on the surface charge [31]. Hassan et al. (2020) showed that EDTA effectively altered the surface charge of carbonate from positive to negative. They noted that the negative value of zeta potential increased with higher concentrations of EDTA and solution pH [32]. Hassan and Al-Hashim (2020) conducted a series of experiments including core flooding, zeta potential analysis, spontaneous imbibition, and NMR. The results of their study revealed that the use of EDTA solution for carbonate core flooding resulted in a shift in wettability towards a more water-wet state [33]. Shi et al. (2020) investigated the effect of the GLDA chelating agent on carbonate samples. They utilized the zeta potential measurement technique, which revealed that GLDA caused a significant shift in the surface charge of the carbonate samples from positive to negative. This change led to an alteration in the rock wettability, resulting in a shift toward a water-wet state [34]. Extensive research has been conducted to assess the impact of smart water and potential determining ions (PDIs) on altering rock wettability and enhancing oil recovery [35]. Rashid et al. (2015) concluded that SO₄^2^⁻ ions play a noteworthy role in modifying rock wettability and act as a catalyst, although their presence is not pivotal for altering rock wettability [36]. Sultani et al. (2012) studied the effects of diverse ions on oil recovery in carbonate reservoirs. Their findings suggested that the presence or absence of SO₄^2^⁻ ions in the injected water would not significantly affect the ultimate oil recovery. However, the incorporation of Mg^2^⁺ ions into the injected water would increase oil recovery by 10–15 % compared to DIW [37]. Karimi et al. (2016) pointed out that enrichment of LSW with Mg^2^⁺ during spontaneous imbibition leads to a decline in ultimate oil recovery [38]. Nevertheless, no study has yet investigated the potential impact of PDIs on the zeta potential of chelating agents.

Despite previous studies on chelating agents, there is still a lack of comprehensive research into the impact of crucial factors on the performance of these agents in altering rock surface charge and changing rock wettability. In this study, we conducted an extensive series of experiments to address this gap. For the first time, we investigated the effects of Potential Determining Ions (PDIs) on the performance of DTPA in altering rock wettability. We examined various reservoir key parameters, including concentration, pH, the presence of oil within the system, Fe^3+^ ions, and salinity, to comprehend how they affect the effectiveness of DTPA chelating agents in altering rock surface charge. DTPA flooding experiments were conducted using an optimal concentration of 5 wt% to observe its notable influence on oil recovery. Finally, we conducted inductively coupled plasma (ICP) analysis on both the SW and DTPA flooding effluents. This technique employs inductively coupled plasma to ionize and measure the concentration of diverse elements within a sample. Our study provides novel insights into the impact of these key parameters on the performance of DTPA chelating agents in oil recovery processes. Understanding these key parameters and optimizing the use of chelating agents can lead to maximizing oil recovery with a minimum cost and without formation damage.

## 2 Experimental work

### 2.1 Materials

#### 2.1.1 Fluids

In this study, different synthetic solutions with varying total dissolved solids (TDS) were used. These solutions were prepared by dissolving pure salts (NaCl, Ca ${\text{Cl}}_{2}$.2 $H_{2}$ O, Mg ${\text{Cl}}_{2}$.6 $H_{2}$ O, ${\text{Na}}_{2}$ S $O_{4}$, and NaHC $O_{3}$) in DIW. The composition of these solutions is detailed in Table 2. Initially, Mg ${\text{Cl}}_{2}$.6 $H_{2}$ O salt was introduced into the DIW due to its relatively high solubility and non-precipitating nature. The solution was then stirred until complete dissolution of the Mg ${\text{Cl}}_{2}$.6 $H_{2}$ O salt was achieved. After confirming the full dissolution of Mg ${\text{Cl}}_{2}$.6 $H_{2}$ O salt, Ca ${\text{Cl}}_{2}$.2 $H_{2}$ O was added, and the solution was again stirred thoroughly to ensure homogeneity. Calcium chloride was selected for its relatively high solubility and compatibility with existing magnesium ions. Following the successful dissolution of magnesium and calcium salts, NaCl, a highly soluble and a major component of natural seawater, was added. Once the previous salts had dissolved completely, ${\text{Na}}_{2}$ S $O_{4}$ was incorporated to contribute to the ionic balance of seawater. Finally, NaHC $O_{3}$ salt was introduced. Continuous monitoring of the solution was crucial to ensure the complete dissolution of each salt before proceeding. Adequate stirring and dissolution time were essential to prevent the formation of precipitates or salt crystals. By following this methodology, uniform preparation procedures were applied to all solutions. In cases where a particular salt was omitted from the solution, the process transitioned to the subsequent steps. According to Table 2, “SW (1 ${\text{Ca}}^{2+}$ + 0 ${\text{Mg}}^{2+}$ + 0 ${\text{SO}}_{4}^{2-})$" represents SW, which ${\text{Mg}}^{2+}$ and ${\text{SO}}_{4}^{2-}$ ions have been eliminated and ${\text{Ca}}^{2+}$ ions are the only PDIs being present in the aqueous phase. Similarly, “SW (3 ${\text{Ca}}^{2+}$ + 0 ${\text{Mg}}^{2+}$ + 0 ${\text{SO}}_{4}^{2-})$" indicates SW with three times of ${\text{Ca}}^{2+}$ concentration, which ${\text{Mg}}^{2+}$ and ${\text{SO}}_{4}^{2-}$ ions have been removed. After preparation, the solutions were stirred for 4 h and filtered through a 0.22 μm filter to remove any remaining solid particles. A solution of 40 wt% of DTPA ($C_{14}H_{18}N_{3}O_{10}K_{5}$) with density of 1.25 g/ ${\text{cm}}^{3}$, molecular weight of 583.8 g/mol and pH of 11 was used. To prepare solutions with lower concentrations of DTPA, such as 1, 3, 5, and 7 wt%, a specific amount of 40 wt% DTPA was dissolved in undiluted SW. At high pH levels, DTPA exhibits enhanced metal ion binding and interaction with reservoir rock surfaces, prompting favorable shifts in wettability and enhanced oil recovery. Therefore, DTPA should be used at a high pH, as it enhances the effectiveness of metal ion chelation [18,35,[39], [40], [41], [42]]. Mixing DTPA with SW caused the pH to decrease, and sodium hydroxide (NaOH) was added to each solution to increase the pH. At high concentrations of DTPA, it was possible to increase the pH to high levels without encountering any precipitation problems, as increasing the DTPA concentration improves its chelation ability. However, at lower concentrations of DTPA, precipitation occurred when the pH of the solution was increased to high levels. Hence, the maximum pH values at which 1, 3, 5, and 7 wt% DTPA solutions remained free of precipitation were 9.48, 10.13, 12.33, and 12.45, respectively.

**Table 2:** Compositions of brine solutions used in this study.

Solution name	Na^+^ (ppm)	Cl^−^(ppm)	K^+^ (ppm)	HCO3^-^(ppm)	Mg^2+^ (ppm)	Ca^2+^ (ppm)	${\text{SO}}_{4}^{2-}$ (ppm)	TDS (ppm)
SW	12649	23287	459	92	1641	499	3069	41696
LSW(0.1SW)	1264	2328	45	9	164	49	306	4165
FW	42467	72904	–	–	1745	1299	–	118415
SW (1 ${\text{Ca}}^{2+}$ +0 ${\text{Mg}}^{2+}$ + 0 ${\text{SO}}_{4}^{2-})$	18384	29607	459	92	0	499	0	49041
SW (3 ${\text{Ca}}^{2+}$ +0 ${\text{Mg}}^{2+}$ + 0 ${\text{SO}}_{4}^{2-})$	17234	29604	459	92	0	1499	0	48888
SW (0 ${\text{Ca}}^{2+}$ +1 ${\text{Mg}}^{2+}$ + 0 ${\text{SO}}_{4}^{2-})$	15856	29615	459	92	1641	0	0	47723
SW (0 ${\text{Ca}}^{2+}$ +3 ${\text{Mg}}^{2+}$ + 0 ${\text{SO}}_{4}^{2-})$	9651	29625	459	92	4925	0	0	44752
SW (0 ${\text{Ca}}^{2+}$ +0 ${\text{Mg}}^{2+}$ + 1 ${\text{SO}}_{4}^{2-})$	17464	25039	459	92	0	0	3069	46123
SW (0 ${\text{Ca}}^{2+}$ +0 ${\text{Mg}}^{2+}$ + 3 ${\text{SO}}_{4}^{2-})$	14477	15898	459	92	0	0	9207	40133

#### 2.1.2 Crude oil

Heavy crude oil from an Iranian oil reservoir was utilized in this study. The oil's properties included an API of 16.97 (at 15.5 °C), a molecular weight of 376.2 g/mol, a specific gravity of 0.953 g/ ${\text{cm}}^{3}$ and viscosity of 80 cp at 80 °C. The SARA analysis results are summarized in Table 3, and the oil composition is listed in Table 4.

**Table 3:** SARA analysis of crude oil.

Component	Weight percent (%)
Saturates	23.55
Aromatic	43.47
Resin	16.58
Asphaltene	16.4

**Table 4:** Composition of crude oil.

component	$C_{2}$	$C_{3}$	$C_{4}$	$C_{5}$	$C_{6}$	$C_{7}$	$C_{8}$	$C_{9}$	$C_{10}$	$C_{11}$	$C_{12+}$
Weight (wt%)	0.01	0.01	0.03	0.26	1.18	0.47	1.92	2.46	1.69	2.37	89.6

#### 2.1.3 Rock samples

Before conducting experiments, it is important to understand the structure of sandstone and the mineral composition of the rock. To achieve this, XRD analysis was employed to identify the rock's characteristics. The sandstone consists of quartz, albite, calcite, magnetite, clinochlore, muscovite, moissanite and microcline. Additionally, XRF analysis was conducted to determine the weight percentage of various elements, especially active cations. The results of the XRF analyses are summarized in Table 5. For wettability experiments, sandstone tablets with a diameter of 3.8 cm and a thickness of 0.2 cm were utilized. Crushed and powdered samples of the rock were also employed for sand pack flooding and zeta potential experiments, respectively.

**Table 5:** Elemental analysis of sandstone using XRF.

Element	Si $O_{2}$	CaO	${\text{Al}}_{2}O_{3}$	${\text{Fe}}_{2}O_{3}$	$K_{2}$ O	${\text{Na}}_{2}$ O	MgO	Ti $O_{2}$	$P_{2}O_{5}$	MnO	SrO	Cl
Weight (%)	47.6	15.10	13.30	7.95	3.64	2.36	2.11	1.21	0.3	0.21	0.2	0.2

### 2.2 Methodology

#### 2.2.1 Wettability measurement

**Fig. 2:**
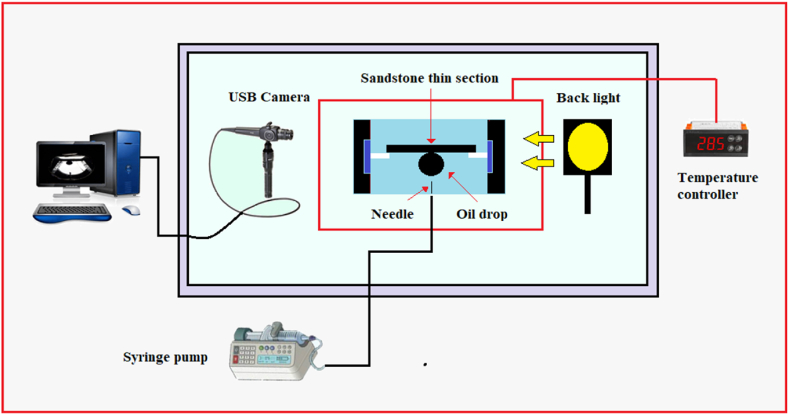
Contact angle set-up.

In this research, we assessed the wettability of sandstone samples using the rock/oil contact angle method. To prepare the sandstone tablets, they were cleaned by washing them with distilled water and dried at 80 °C for 24 h to remove any dust. For the wettability alteration experiments, the sandstone tablets were initially placed in FW at 80 °C for one week, and the rock/oil contact angle was measured as the initial water-wet angle (${ϴ}_{i}$) for all samples. Subsequently, the samples were immersed in crude oil at 80 °C for 21 days, and the rock/oil contact angle was measured as the oil-wet angle at zero time (${ϴ}_{0}$). To investigate the effect of DTPA on the wettability of the rock, sandstone tablets were immersed in different concentrations of DTPA, and the contact angle ${ϴ}_{f}$ between the rock and oil was measured at different time intervals (2, 4, 8, 12, 24, 48, 72, 120, 240, 360, and 504 h) using Image-J analysis software. It should be noted that, to minimize topography effects, multiple oil droplets were placed on both sides of the rock tablet during each contact angle measurement. Fig. 2 illustrates the setup used for contact angle measurements.

#### 2.2.2 Zeta potential measurements

Zeta potential measurements were employed to assess the surface charge of the rock samples. The impact of the DTPA chelating agent on the zeta potential was evaluated through the following procedures:

1. Zeta potential measurement in absence of oil:

To investigate the zeta potential at the brine/rock interface in the absence of oil, a series of solutions were prepared. Each solution comprised 1 wt% sandstone powder dissolved in different concentrations of DTPA (1 wt%, 3 wt%, 5 wt% and 7 wt%) in SW.

2. Zeta potential measurement in the presence of oil:

Similarly, to examine the zeta potential at the brine/rock interface in the presence of oil, the same solutions as in the previous step were used. However, this time, 1 wt% crude oil was introduced into these solutions.

Once the solutions from steps (1) and (2) were prepared, they were shaken using a shaker for 24 h at 30 °C and atmospheric pressure. Following this period, the samples were removed from the shaker and allowed to settle for 30 min. Subsequently, a homogeneous sample was taken from the top of each solution using a syringe and then filtered through a 5 mm filter.

Finally, the zeta potential was measured using a Brookhaven Zeta PALS instrument, and the Smoluchowski equation (equation (3)) was employed for data analysis [43].

(3) $$\zeta =113000\;\text{EM}\frac{V_{t}}{D_{t}}$$

Where ***ζ***: zeta potential (mV), *v*_*t*_: viscosity of the suspending liquid at temperature t (poise), *D*_*t*_: dielectric constant of the suspending liquid at temperature t, EM: electrophoretic mobility at actual temperature (μm.s^−1^.V^−1^.cm) [43].

#### 2.2.3 Sand pack flooding

**Fig. 3:**
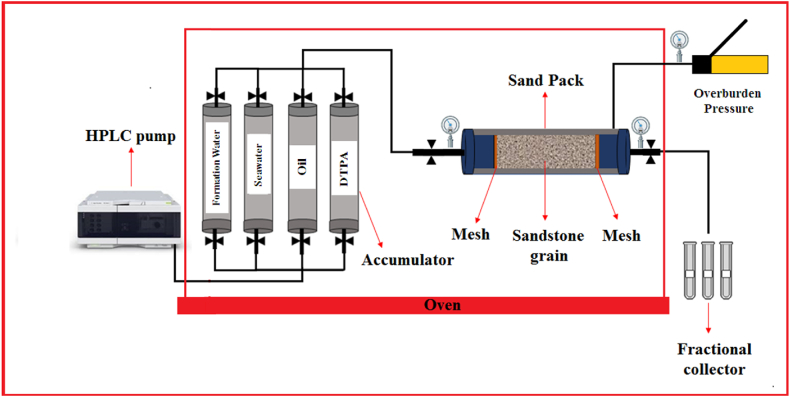
Flooding system.

The sand pack flooding test was conducted by injecting SW along with a 5 wt% DTPA chelating agent. The experimental setup included a sand pack holder with dimensions of 6 cm in diameter and 11 cm in length, an oven, differential pressure transducers, an HPLC pump, transfer cylinders (accumulators), and outlet collectors for collecting flooding effluents. The sandstone was crushed to a size of 20–45 μm and packed into the sand pack holder. To prevent sand from flowing out and to ensure an even distribution of the injected fluid, two 200-μm mesh screens were positioned at the inlet and outlet of the sand pack. Different accumulators were used for injecting the liquids into the sand pack. Porosity was measured, and permeability was determined at three different rates using differential pressure transducers and the Darcy equation [44]. Establishing the initial water saturation required injecting oil into the rock and allowing it to age. In our study, heavy dead oil was introduced into the sand pack and allowed to age for three weeks. Subsequently, sand pack flooding was carried out by injecting SW and 5 wt% DTPA at a temperature of 80 °C, a pressure of 1000 psi, and a flow rate of 0.5 cc/m^3^. Table 6 provides a summary of the petrophysical properties of the sand pack, and Fig. 3 illustrates the flooding setup.

**Table 6:** Properties of the sand pack.

Sand pack length (cm)	Sand pack diameter (cm)	Porosity (%)	Permeability (D)	Pore volume (cc)	Bulk volume (cc)	Swi (%)
11	6	31	3.4	60.97	311.01	35

**Table 7:** WAI for different solutions.

Number	solutions	${ϴ}_{i}$	${ϴ}_{0}$	${ϴ}_{f}$	WAI
1	SW	28	160	105	0.4
2	LSW	24	152	36	0.9
3	1 wt% DTPA/SW	30	138	93	0.41
4	3 wt% DTPA/SW	28	141	82	0.52
5	5 wt% DTPA/SW	22	143	23	0.99
6	7 wt% DTPA/SW	27	153	22	1.03
7	5 wt% DTPA/DIW	25	148	17	1.06

## 3 Results and discussion

### 3.1 Wettability alteration

**Fig. 4:**
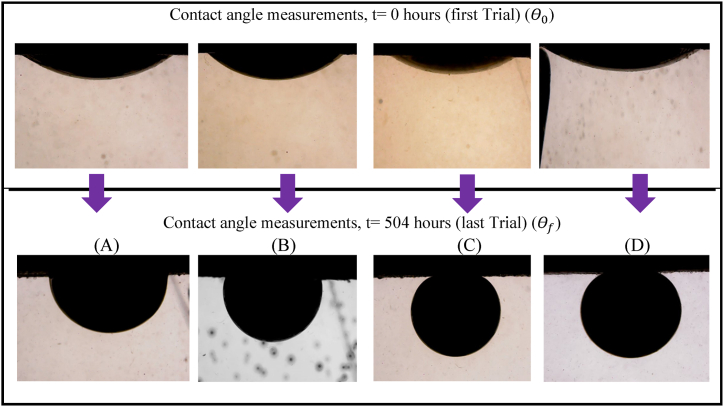
Crude oil droplets in contact with thin sections and different solutions; (A) 1 wt% DTPA/SW, (B) 3 wt% DTPA/SW, (C) 5 wt% DTPA/SW, and (D) 7 wt% DTPA/SW.

**Fig. 5:**
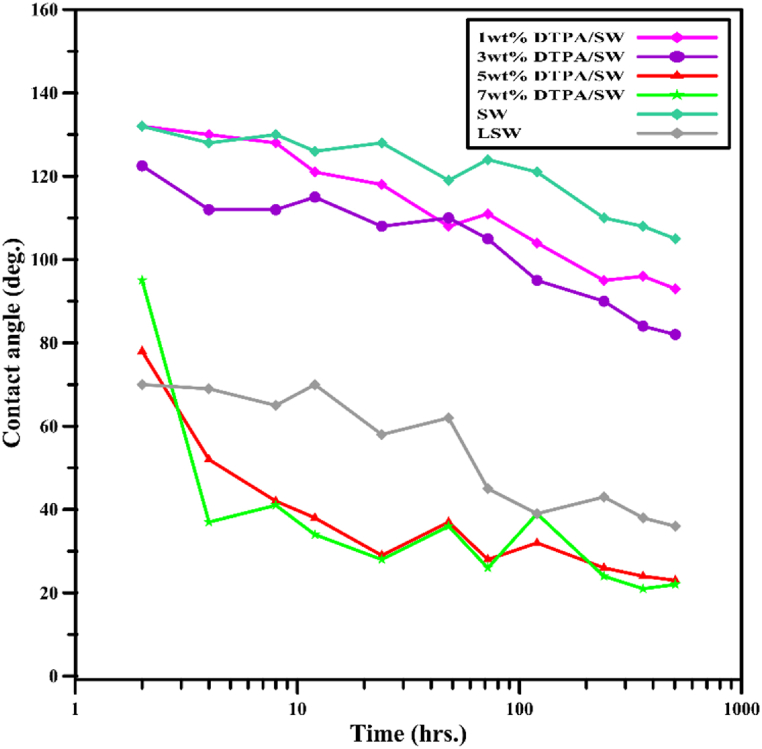
The effect of different solutions on rock/oil contact angle over time.

A DTPA chelating agent with an initial concentration of 40 wt% was diluted in raw SW to prepare solutions containing 1 wt%, 3 wt%, 5 wt%, and 7 wt% DTPA. These solutions were used to investigate the effect of DTPA chelating agents on wettability alteration. Fig. 4(A–D) shows the oil droplets on the sandstone tablets that were exposed to various solutions and aged. It can be observed that increasing DTPA concentration led to a more water-wet wettability of the sandstone tablets. Fig. 5 also shows the wettability alteration of sandstone in contact with different solutions over time. As can be seen, the 1 wt% and 3 wt% DTPA solutions did not completely shift the wettability to water-wet. In contrast, the 5 wt% and 7 wt% DTPA solutions significantly altered the rock/oil contact angle, reducing it from 143° to 153°–23° and 22°, respectively. In fact, as the concentration of DTPA increases, the chelation force also increases, allowing more metal ions to be chelated from both the rock and solution. It was observed that when the DTPA concentration is below 5 wt%, it may not have sufficient strength to chelate all the metal ions from the rock and solution. Therefore, higher concentrations should be employed. Increasing DTPA concentration from 3 wt% to 5 wt% induced a quick shift in wettability from oil-wet to water-wet. This shift can be attributed to several factors: firstly, at 3 wt%, not all rock surface adsorption sites are saturated, resulting in moderate wettability change. However, at 5 wt%, more binding sites on the rock surface become occupied, strengthening surface interactions. Secondly, higher DTPA concentrations can displace previously adsorbed substances, leading to wettability alteration [45]. Lastly, as described below, 5 wt% DTPA acts as a threshold concentration, significantly accelerating the wettability change process, making it effective in displacing oil with water [46]. On the other hand, as the DTPA concentration was increased from 5 to 7 wt%, the wettability did not undergo significant changes. In fact, 5 wt% DTPA was found to be sufficient for chelating cations from the rock and solution, and elevating the DTPA concentration up to 7 wt% did not yield substantial changes in wettability. This phenomenon is attributed to the finite number of binding sites at the surface. When chelating agents come into contact with rock surfaces the availability of binding sites on the surface can influence the extent of chelation. When concentrations of these agents become substantial, saturation can occur. This saturation point indicates that the maximum number of DTPA to form complexes has been attained. Consequently, the introduction of additional DTPA into the system at this point does not lead to further sequestration of multivalent cations. As a result, there would be no significant change in the wettability alteration [[46], [47], [48]]. While chelating agents like DTPA remove metal ions from the solution and affect wettability indirectly, direct interactions with rock surfaces also occur, modifying surface properties. Both mechanisms are intertwined, complicating the isolation of their individual effects on wettability alteration. Comparing the wettability alteration of samples placed in 5 wt% DTPA and LSW solutions revealed that 5 wt% DTPA performed similarly to LSW. In fact, 5 wt% DTPA solution results in a more pronounced rock wettability alteration compared to LSW. Therefore, employing a DTPA chelating agent at an appropriate concentration and pH can serve as an effective alternative to LSW for wettability alteration. This approach eliminates the cost of SW dilution and prevents the problems that can arise from using LSW. To evaluate the direct impact of DTPA on the rock, a 5 wt% DTPA solution was prepared using DIW, and its effect on rock wettability alteration was investigated. It is evident that DTPA directly targets metal ions on the rock surface, leading to enhanced chelation and, consequently, greater wettability alteration compared to alternative solutions. Nevertheless, the expenses associated with the preparation of DIW should be taken into account. Furthermore, the results demonstrated that a decrease in salinity from SW to LSW (a 10-times diluted SW) resulted in wettability alteration from oil-wetness to water-wetness (105°–36°, respectively).

Table 7 presents the Wettability Alteration Index (WAI) for various solutions. The WAI serves as a measure of the performance of the DTPA chelating agent in modifying the rock surface charge. The WAI is calculated using equation (4):

(4) $$\text{WAI}=\frac{{ϴ}_{0}-{ϴ}_{f}}{{ϴ}_{0}-{ϴ}_{i}}$$

Where ${ϴ}_{i}$ represents the initial rock/oil contact angle before aging sandstone tablets in oil, ${ϴ}_{0}$ represents the rock/oil contact angle after aging the tablets in oil, and ${ϴ}_{f}$ represents the final rock/oil contact angle after immersing the tablets in the designated solutions. A WAI value nearing zero indicates a negligible change in wettability, while a value approaching one suggests water-wet wettability.

### 3.2 Zeta potential

**Fig. 6:**
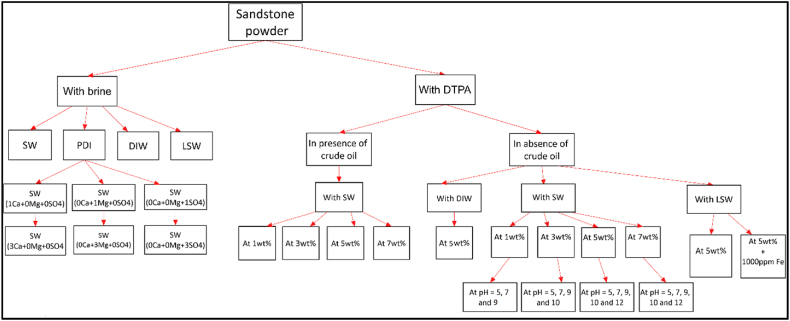
Summary of the zeta potential experiments.

When the clay surface is exposed to an aqueous solution, a stern layer consisting of a thin layer of cations forms on the clay surface. In addition, a diffuse layer of ions with opposite polarity, which is thicker, also forms. This combined structure of the stern layer and the diffuse layer is known as the electrical double layer, with a reported thickness of 0.01 μm [49]. The zeta potential, which measures the potential in the electrical double layer, is used to determine the rock surface charge [42]. When the charges at the oil/brine and rock/brine interfaces are the same, repulsive forces are generated, causing the water film to expand around the rock surface [50]. This increase in water film thickness allows oil droplets to easily separate from the rock surface, leading to improved oil recovery [[51], [52], [53], [54]]. Various models, such as the Gouy-Chapman model, have been proposed to describe the electrical double layer [55]. This model shows the relation between zeta potential and the electric double layer thickness [56]. A positive zeta potential indicates oil-wetness, while a negative zeta potential indicates water-wetness of the rock [57]. Studies have shown that increasing the negative value of zeta potential can make the rock surface more water-wet, resulting in improved oil recovery [32,39,58]. Chelating agents, such as DTPA, can increase the water film thickness around the rock surface by increasing the negative charge at the rock/brine and oil/brine interfaces. Furthermore, the use of chelating agents at high pH levels can increase the negative value of zeta potential, thereby improving oil recovery. The relationship between the chelation of various ions using chelating agents and the increase of negative zeta potential values lies in the electrostatic repulsion phenomena at the fluid-rock interface. Chelating agents possess the ability to form stable complexes with metal ions present in the solution and rock. As these metal ions are bound by the chelating agent, their potential to adsorb onto the rock surface is reduced. This, in turn, leads to a higher negative charge density on the rock surface. The negative zeta potential of the rock surface reflects its overall charge and wettability characteristics. An increased negative zeta potential indicates enhanced electrostatic repulsion between the negatively charged rock surface and the reservoir fluid. This electrostatic repulsion counteracts the forces that typically hinder fluid penetration into the rock matrix, facilitating oil detachment and mobilization. Therefore, the rise in negative zeta potential values is directly linked to the chelation of ions by chelating agents. By forming complexes with metal ions, these agents prevent these ions from being available for adsorption onto the rock surface. Consequently, the overall charge balance at the fluid-rock interface shifts towards negativity, resulting in the observed increase in the negative zeta potential values. The aim of this section is to study the impact of DTPA chelating agent on the sandstone surface charge. To achieve this, sandstone powder was mixed with various DTPA solutions and the pH of these solutions was adjusted using HCl and NaOH. To ensure reproducibility, at least three measurements were conducted for each sample, and the zeta potential values were reported. Fig. 6 provides a summary of the zeta potential experiments.

#### 3.2.1 Effect of concentration

**Fig. 7:**
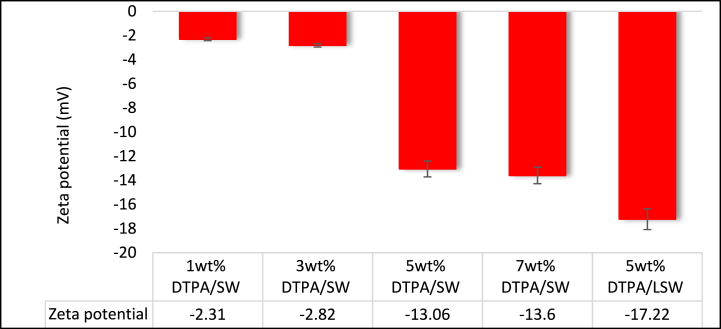
Zeta potential for sandstone and different concentrations of DTPA chelating agent.

Altering the rock surface charge from positive to negative, or from low negative to high negative, can result in a decrease in residual oil saturation and a shift in the rock wettability from oil-wet to water-wet [39]. Solutions of 1, 3, 5, and 7 wt% DTPA were used to investigate the effect of DTPA concentration on the rock surface charge. It was observed that as the concentration of DTPA chelating agent increased, the chelation force became stronger, resulting in the chelation of metal ions from both the fluid and the rock. This chelation process led to an increase in the thickness of the water film and the electrical double layer on the rock surface, causing the zeta potential, which reflects the surface charge, to become more negative. Consequently, the oil was more easily separated from the rock surface due to the enhanced repulsion between the negatively charged rock surface and the oil phase [58]. Fig. 7 illustrates the zeta potential measurements for sandstone at various concentrations of DTPA. Notably, all DTPA concentrations resulted in a negative zeta potential, and increasing DTPA concentration led to a more negative zeta potential and a shift towards more water-wetness in terms of wettability. This suggests that the higher DTPA concentration promoted a stronger repulsion of oil, facilitating the transition of rock wettability from oil-wet to water-wet, which can have significant implications in oil recovery processes.

The zeta potential values were measured for sandstone samples treated with 1, 3, 5, and 7 wt% DTPA solutions and were found to be −2.31, −2.81, −13.06, and −13.6 mV, respectively. Notably, 1 and 3 wt% DTPA concentrations resulted in only slight changes in the zeta potential values of the sandstone, suggesting that the rock surface maintained a relatively neutral charge. However, as the DTPA concentration increased to 5 wt%, the solution exhibited enhanced reactivity with the present salts, leading to a significant decrease in the zeta potential. This suggests that DTPA molecules were able to effectively chelate the metal ions from both the rock surface and the solution, resulting in a greater negative charge on the rock surface. Interestingly, further increasing the DTPA concentration from 5 to 7 wt% did not cause a noticeable change in the zeta potential. This can be attributed to the fact that, at 5 wt% the surface was already saturated with DTPA molecules, leaving no available sites for further interaction with metal ions. Consequently, the chelation force remained constant, resulting in minimal changes in zeta potential at higher concentrations. Therefore, 5 wt% DTPA solution is sufficient to chelate metal ions from both rock and solution, and increasing DTPA concentration beyond this point does not contribute to further increase in the negative charge of the rock. Hence, 5 wt% DTPA solution was determined as the optimal concentration in this study.

The presence of active ions in SW can have a significant impact on the chelation force and zeta potential and reduces the negative values of zeta potential. For instance, as illustrated in Fig. 7, when 5 wt% DTPA solution was prepared using SW, the zeta potential measured −13.06 mV. In contrast, substituting SW with LSW resulted in an increased negative zeta potential value of −17.22 mV. This observation indicates that the presence of active ions in SW diminishes the chelation force of DTPA. Conversely, the lower concentration of various ions in LSW does not affect the chelation force of DTPA when it is prepared in LSW. It should be noted that there may be additional costs associated with the use of LSW compared to SW in EOR operations. LSW may require additional treatment or transportation costs to reduce its salinity to an optimal level for EOR operations. Therefore, economic considerations should be taken into account when selecting the appropriate water type for EOR operations [30].

#### 3.2.2 Effect of salinity

Due to its high concentration of multivalent cations, SW tends to reduce the negative value of zeta potential and can even shift the surface charge to become neutral or positive. This phenomenon arises from the ability of multivalent cations to compress the electrical double layer, resulting in more positive surface charge values. Trivalent cations, such as Al^3+^, exert a greater influence on the surface charge of rocks compared to divalent cations. For instance, Al^3+^ can compress the electrical double layer more effectively than Ca^2+^ leading to a more positive charge on the kaolinite surface [59]. In clay sandstone reservoirs, using high salinity water is not recommended for enhancing oil recovery. This is because high salinity and high concentrations of divalent cations can cause precipitation and reduce the thickness of the electrical double layer. Chen et al. (2014) reported that salinity is a factor affecting the zeta potential. With increasing solution salinity, the zeta potential tends to become more positive [60]. According to Ramez et al. (2011), increasing the salinity of the solution can decrease the negative value of zeta potential, resulting in lower oil recovery. Conversely, lowering the salinity of solution can increase the negative value of zeta potential and lead to higher oil recovery [61].

One of the models proposed to describe zeta potential is the Gouy-Chapman model [55], presented in equations (5), (6). These equations illustrate that in high salinity solutions, the thickness of the electrical double layer decreases due to the abundance of multivalent cations and high salt concentrations. This reduction causes an increase in the zeta potential. Nevertheless, chelating agents have the capacity to capture and bind multivalent cations from the solution. As a result, the salinity decreases and the electrical double layer thickness expands.

(5) $$\frac{1}{K}=\left(\frac{\varepsilon_{0}\varepsilon kT}{2n_{0}e^{2}v^{2}}\right)$$

(6) ζ=2KTvesinh−1[σe(1k)4n0ve]

where ***ζ***: zeta potential value (mV), T: temperature (K), K: Boltzmann constant (J K^−1^), e: electrical charge (C), *v:* cation valence, $\sigma_{e}$: electrokinetic charge density (C M^−2^), $n_{0}$: ion concentration (m^−3^), $\frac{1}{K}$: double layer thickness (m), $\varepsilon_{0}$: vacuum dielectric permittivity (F M^−1^), ε: water dielectric constant.

**Fig. 8:**
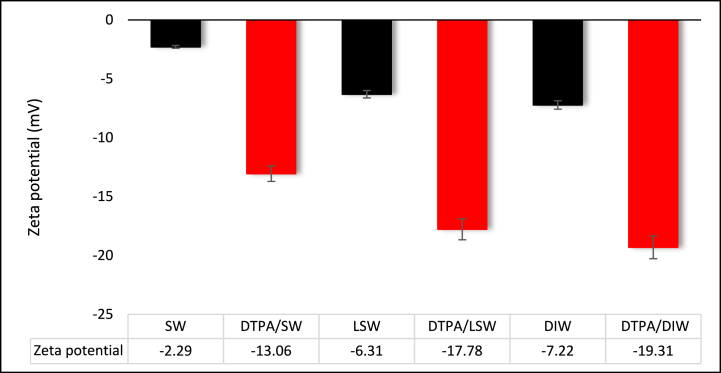
Zeta potential for sandstone and different solutions.

To investigate the influence of salinity on zeta potential, three distinct solutions, SW, LSW, and DIW, were employed. These solutions, with pH levels ranging from 7 to 8, were used to measure the zeta potential for sandstone Fig. 8 displays the zeta potential values at different salinities. As demonstrated, the zeta potential of SW exhibits a lower negative value compared to LSW, while DIW displays a more negative zeta potential than LSW. The specific values for zeta potential are as follows: 2.29 mV for SW, −6.31 mV for LSW, and −7.22 mV for DIW. SW contains a notable concentration of positive ions that hinder surface dissolution, resulting in a positive zeta potential. Consequently, sandstone rocks subjected to high salinity solutions tend to have either a low negative or positive surface charge. However, DIW has a more negative surface charge due to the absence of cations in its composition. To assess the performance of the DTPA chelating agent within different saline solutions, 40 wt% DTPA solution was diluted in various saline solutions and solutions of 5 wt% DTPA/SW, 5 wt% DTPA/LSW, and 5 wt% DTPA/DIW were prepared. Subsequently, the zeta potential of sandstone and these solutions was measured. It is worth noting that the DTPA/DIW solution was utilized to examine the direct effect of DTPA on the rock surface.

While all samples exhibited negative zeta potential values, the presence of various cations in aqueous solutions exerted an influence on the chelation force of DTPA. In fact, the efficacy of the DTPA chelating agent to sequester metal ions and modify rock surface charge is impaired in high salinity solutions due to the large number of multivalent cations. The results of zeta potential measurements showed that DTPA/SW reduced the zeta potential from −2.29 to −13.06 mV, while introducing DTPA in LSW decreased the zeta potential from −6.31 to −17.75 mV. Moreover, DTPA chelating agent prepared in DIW (DTPA/DIW) altered the zeta potential from −7.22 to −19.31 mV. DTPA directly interacts the rock surface and chelates metal ions due to the absence of ions in DIW, resulting in a more pronounced negative zeta potential value. Compared to SW and LSW solutions, DTPA has an advantage in effectively chelating metal ions from both solution and rock, thereby increasing the negative value of zeta potential.

#### 3.2.3 Effect of potential determining ions (PDIs)

The role of zeta potential in understanding the impact of ions on rock wettability alteration is crucial. Divalent ions, including ${\text{Ca}}^{2+}$, ${\text{Mg}}^{2+}$, and ${\text{So}}_{4}^{2-}$ possess the capacity to substantially modify the zeta potential of rock surfaces, leading to changes in rock wettability and ultimately influencing oil recovery processes. Extensive research has been conducted to understand the impact of smart water and various ions on rock wettability alteration and oil recovery. In this study, we have examined the effects of ${\text{Ca}}^{2+}$, ${\text{Mg}}^{2+}$, and ${\text{So}}_{4}^{2-}$ ions on the performance of the DTPA chelating agent. These ions were selected due to their potential significance and their considerable presence in the SW. Furthermore, several studies have emphasized that the presence of ${\text{Ca}}^{2+}$, ${\text{Mg}}^{2+}$, and ${\text{So}}_{4}^{2-}$ in the injected water plays a crucial role in altering the rock surface charge [62]. Divalent ions, such as ${\text{Ca}}^{2+}$, ${\text{Mg}}^{2+}$, and ${\text{So}}_{4}^{2-}$ exert a more pronounced influence on rock wettability when compared to monovalent ions. This effect is attributed to their high charge density and capacity, enabling them to effectively displace polar components from the rock surface. In order to assess the performance of chelating agents SW, it is necessary to conduct all tests under the same ionic strength as SW. To achieve this, the concentration of NaCl in the solution was adjusted each time by adding or reducing ${\text{Ca}}^{2+}$, ${\text{Mg}}^{2+}$, and ${\text{So}}_{4}^{2-}$ ions to maintain the ionic strength at the same level as SW.

**Fig. 9:**
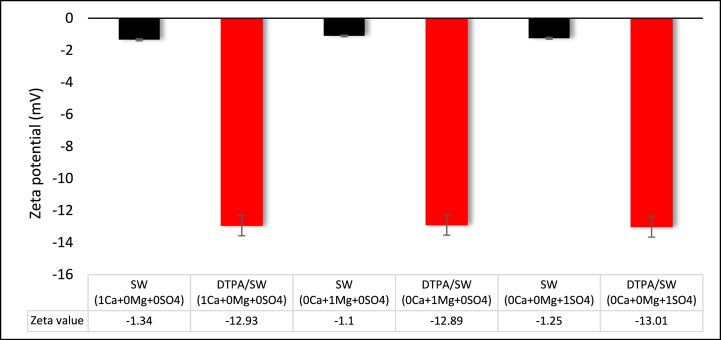
Effect of PDI on the zeta potential of sandstone and DTPA solutions.

As shown in Fig. 9, zeta potential for sandstone and SW (1 ${\text{Ca}}^{2+}$ +0 ${\text{Mg}}^{2+}$ +0 ${\text{SO}}_{4}^{2-}$) was −1.34. However, upon introducing a 5 wt% DTPA chelating agent to this brine, the negative value of zeta potential significantly improved and changed to −12.93. The capture of metal ions from the solution using DTPA causes the rock to release cations from its surface. This process changes the rock surface charge by releasing cations into the solution. When ${\text{Ca}}^{2+}$ and ${\text{SO}}_{4}^{2-}$ ions were eliminated from SW, the zeta potential became −1.10. However, diluting 5 wt% DTPA in this solution changed the zeta potential from −1.10 to −12.89, inducing a water-wet condition. Furthermore, by eliminating ${\text{Ca}}^{2+}$ and ${\text{Mg}}^{2+}$ ions from SW, the zeta potential shifted to −1.25. Subsequently, the introduction of DTPA to this solution resulted in a zeta potential alteration from −1.25 to −13.01, leading to a transition in rock wettability from a slight oil-wetness to a strong water-wetness. Therefore, introducing the DTPA chelating agent to SW, in the presence or absence of any potential determining ions, significantly increases the negative amount of zeta potential and changes the rock wettability from oil-wet to strongly water-wet. Based on Fig. 9, we can conclude that the presence or absence of potential determining ions such as ${\text{Ca}}^{2+}$, ${\text{Mg}}^{2+}$, and ${\text{SO}}_{4}^{2-}$ in the solution has no effect on the efficacy of 5 wt% DTPA chelating agent. DTPA captures and chelates metal ions, preventing their interaction with other ions. Interestingly, samples containing DTPA chelating agents exhibit the same zeta potential. Therefore, the type of ion does not impact the performance of DTPA, and the only difference may arise from variations in the stability constants of DTPA with different ions. DTPA prioritizes ions with higher stability constants before attacking those with lower stability constants.

**Fig. 10:**
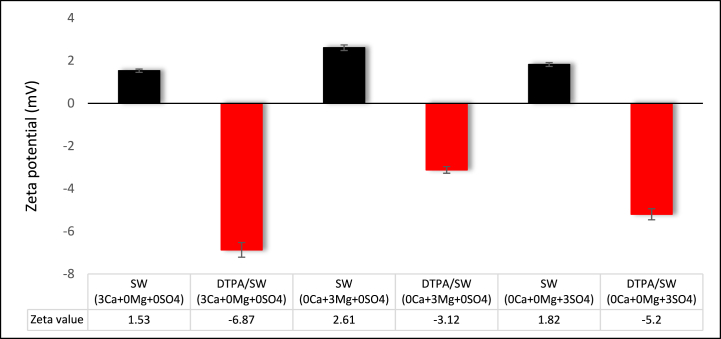
Effect of tripling the concentration of PDI on the zeta potential of sandstone and DTPA solutions.

Fig. 10 illustrates that tripling the concentration of ${\text{Ca}}^{2+}$, ${\text{Mg}}^{2+}$, and ${\text{SO}}_{4}^{2-}$ has led to an increase in the zeta potential. The chelation force decreases with increasing PDI concentration in the solution so that 5 wt% DTPA is not sufficient to chelate all PDIs from the solution. Comparing samples sandstone/DTPA/SW (3 ${\text{Ca}}^{2+}$ + 0 ${\text{Mg}}^{2+}$ + 0 ${\text{SO}}_{4}^{2-})$, sandstone/DTPA/SW (0 ${\text{Ca}}^{2+}$ + 3 ${\text{Mg}}^{2+}$ + 0 ${\text{SO}}_{4}^{2-})$ and sandstone/DTPA/SW (0 ${\text{Ca}}^{2+}$ + 0 ${\text{Mg}}^{2+}$ + 3 ${\text{SO}}_{4}^{2-})$ reveals that the zeta potential for sandstone/DTPA/SW (0 ${\text{Ca}}^{2+}$ + 3 ${\text{Mg}}^{2+}$ + 0 ${\text{SO}}_{4}^{2-}$) is higher than the other two samples, owing to the lower concentration of ${\text{Na}}^{+}$ in compared to the other two samples. According to Table 2, the ${\text{Na}}^{+}$ ion concentration in the sample containing SW(0 ${\text{Ca}}^{2+}$ +3 ${\text{Mg}}^{2+}$ + 0 ${\text{SO}}_{4}^{2-}$) is 9651 ppm, whereas this value is 17234 and 14477 ppm for the samples containing SW(3 ${\text{Ca}}^{2+}$ +0 ${\text{Mg}}^{2+}$ + 0 ${\text{SO}}_{4}^{2-})$ and SW(0 ${\text{Ca}}^{2+}$ +0 ${\text{Mg}}^{2+}$ + 3 ${\text{SO}}_{4}^{2-}$), respectively. In clay surfaces, ${\text{Na}}^{+}$ ions in the aqueous phase replace divalent cations [62]. In fact, as metal ions are chelated from the solution, ${\text{Ca}}^{2+}$ and ${\text{Mg}}^{2+}$ ions are separated from the rock surface to establish equilibrium, and ${\text{Na}}^{+}$ ions replace them on the clay surface. In the case of sandstone/DTPA/SW (0 ${\text{Ca}}^{2+}$ + 3 ${\text{Mg}}^{2+}$ + 0 ${\text{SO}}_{4}^{2-}$), which has a low concentration of ${\text{Na}}^{+}$ ions in its structure, cation substitution does not occur effectively. Moreover, when the ion concentration is varied from one to three times, 5 wt% DTPA is unable to chelate all the metal ions from the solution and rock, resulting in a weak change in rock wettability towards water-wet conditions. As a result, the negative value of zeta potential for sandstone/DTPA/SW (0 ${\text{Ca}}^{2+}$ + 3 ${\text{Mg}}^{2+}$ + 0 ${\text{SO}}_{4}^{2-}$) is lower than that of the other two solutions. However, it is noteworthy that a high concentration of ${\text{Na}}^{+}$ ions in the solution reduces the activity of divalent ions and hinders their access to the electrical double layer. On the other hand, the injection of sulfate ions into the water reduces the cation concentration in the solution, leading to a significant increase in the negative charge density near the clay surface. This disrupts the initial equilibrium between the rock and the FW due to the disparity in cation concentrations. As a result, divalent cations such as ${\text{Mg}}^{2+}$ and ${\text{Ca}}^{2+}$ are released from the clay surface, whereas ${\text{Na}}^{+}$ ions are adsorbed onto the rock surface, causing the surface to become water-wet.

#### 3.2.4 Effect of iron ions

**Fig. 11:**
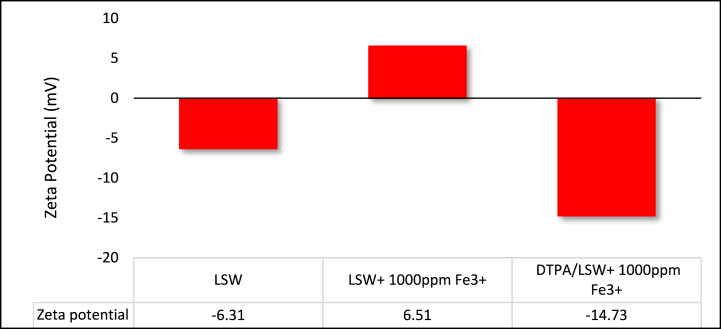
Effect of iron ions on sandstone surface charge.

In oil reservoirs containing iron minerals, the presence of iron ions can lead to a positive zeta potential value, significantly influencing the oil recovery process. The removal of iron ions from the rock or the pH adjustment of the solution in such reservoirs can enhance oil recovery by modifying the zeta potential. Iron ions have the capacity to shift the zeta potential from a highly negative value to a less negative, or even a positive, value. Therefore, compared to other clay minerals, chlorite minerals (which contain iron ions) exhibit a lower zeta potential [52]. Chelating multivalent cations can eliminate the effect of iron ions, as these ions play a pivotal role in balancing the sandstone surface charge and increasing the negative zeta potential value [63]. Chelating agents separate multivalent cations, such as iron ions, from both rocks and solutions. This separation increases the negative charge on the Mg–OH and Si–OH surfaces [[64], [65], [66]]. They can simultaneously increase the pH of injected fluids and remove free iron ions from the solution. This elimination of iron minerals, whether by increasing the pH of the solution or by removing iron ions from the rock and solution, has been found to make the rock surface more negative. This process enhances oil recovery from sandstone reservoirs containing iron minerals [4,67]. Asphaltenes are colloidal particles dispersed in crude oil that can precipitate when asphaltene micelles are disrupted [68]. Iron ions have been identified as contributing factors to asphaltene precipitation, and chelating agents can prevent this by capturing and chelating these ions [69,70]. The impact of Fe^3+^ ions on the surface charge of the rock was analyzed in this study. As illustrated in Fig. 11, the zeta potential for sandstone powder and LSW was measured at −6.31 mV. However, upon adding 1000 ppm Fe^3+^ to LSW, the zeta potential increased to +6.51 mV. This suggests that iron ions can alter the wettability of sandstone, shifting it from water-wet to oil-wet. By diluting DTPA in this solution (5 wt% DTPA/LSW+1000 ppm Fe^3+^), the zeta potential changed from +6.51 to −14.73 mV. The addition of the DTPA chelating agent to the injected solution is beneficial, as it transforms the rock wettability from oil-wet to strongly water-wet. DTPA chelates iron ions from the rock, and if the rock is exposed to DTPA at high temperatures for an extended period, it can lead to the chelation of all iron and metal ions. Therefore, chelating agents have the potential to alter the mineralogy of the rock, shifting its surface charge from positive to negative.

#### 3.2.5 Presence of crude oil

The charge at oil/brine and rock/brine interfaces indicates the wettability status of reservoirs. The negative charge increases the repulsive forces between oil droplets and the rock surface, releasing oil from the rock surface. Studies have indicated that in the presence of crude oil, negatively charged polar components are adsorbed onto the carbonate rock surface, leading to an increased release of calcium ions from the rock. Consequently, the presence of crude oil results in heightened negative values of zeta potential [54,71,72].

**Fig. 12:**
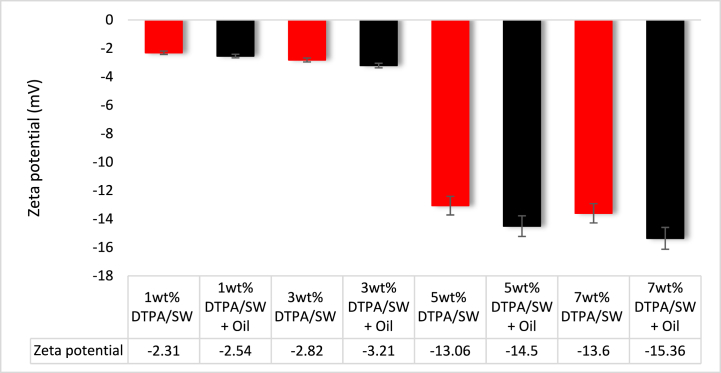
Effect of crude oil on the zeta potential of sandstone and DTPA solutions.

The effect of crude oil on the surface charge of the DTPA/sandstone system was investigated. Initially, DTPA/sandstone samples were prepared and the zeta potential was measured for each sample. Subsequently, in order to assess the impact of oil on DTPA performance and zeta potential, 1 wt% oil was added to 1, 3, 5, and 7 wt% DTPA solutions, and the zeta potential was measured for sandstone/DTPA/oil samples. It should be noted that the DTPA solutions were prepared using SW. The impact of crude oil on the zeta potential for sandstone/DTPA/SW samples was shown in Fig. 12. All the samples exhibited negative zeta-potential values. Moreover, an increase in the concentration of DTPA in the sandstone/oil system resulted in a more negative surface charge. For DTPA solutions with concentrations of 1, 3, 5, and 7 wt% in the absence of crude oil, the zeta potential values were −2.31, −2.82, −13.06, and −13.6 mV, respectively. However, with the presence of crude oil, these values decreased to −2.54, −3.21, −14.5, and −15.36 mV, respectively. Consequently, the samples containing crude oil exhibited a more negative surface charge compared to those prepared without crude oil, with zeta potential values being 10 %–15 % more negative. The reduction in zeta potential in the presence of crude oil can be attributed to the presence of carboxylic groups in the hydrocarbon phase of the crude oil [51,73]. In crude oil, carboxylic groups are commonly found in the form of carboxylic acids, which are organic acids containing one or more carboxyl groups (COOH). When crude oil is introduced into the sandstone/DTPA/SW system, the carboxylic groups present in the crude oil can adsorb onto the surface of the particles within the system. Once adsorbed, these carboxylic groups are likely to ionize, meaning they can dissociate into negatively charged ions (COO-) within the system. These negatively charged ions can then interact with the particles, leading to an increase in the negativity of the surface charge of the particles. This increased negativity of the surface charge results in more negative zeta potential values. Therefore, the adsorption of crude oil and the presence of carboxylic groups cause the surface charge of samples containing crude oil to become more negative compared to those without crude oil.

#### 3.2.6 Effect of pH

Numerous studies have highlighted the substantial impact of pH values on contact angle measurements, indicating that the rock/oil contact angle tends to decrease as pH increases [61]. Fluids with higher pH levels are recognized for their ability to shift rock wettability from oil-wetness towards water-wetness. Al-Rossies et al. (2010) observed that when brine with pH values above 6.5 is present, sand grain wettability remains water-wet, whereas pH values below 4.4 lead to oil-wet wettability. This behavior is also influenced by the composition of the crude oil [74]. The introduction of chelating agents to the FW increases the pH of the solution, generating a repulsive force between the rock and oil. Consequently, this mechanism enhances oil recovery through wettability alteration.

**Fig. 13:**
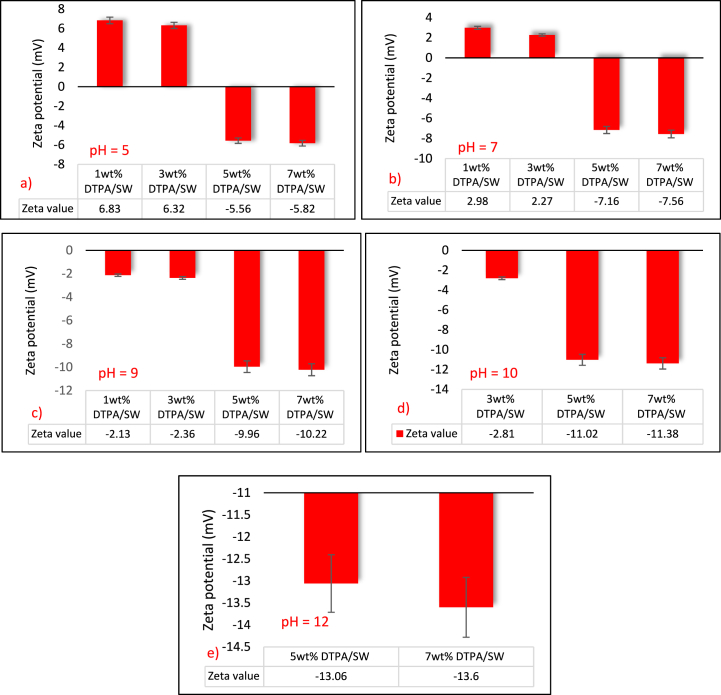
Zeta potential for sandstone and DTPA/SW solution at acidic and basic pH.

The influence of pH on rock surface charge was investigated using four different concentrations of DTPA, ranging from 1 to 7 wt%, and by varying the solution pH from 5 to 12. Fig. 13(a - e) displays the zeta potential results for sandstone when exposed to the four DTPA solutions. As shown in the figure, zeta potential values consistently display negativity across all pH levels for 5 and 7 wt% DTPA concentrations. Moreover, an increase in pH induces a shift in the zeta potential from a low negative value to a high negative one. On the other hand, concentrations of 1 and 3 wt% DTPA exhibit positive zeta potential at lower pH levels, while showing negative zeta potential at higher pH levels. Furthermore, under the same pH conditions, an increase in DTPA concentration leads to a reduction in the zeta potential value. Therefore, DTPA demonstrates good performance in improving the wettability condition at higher concentrations and pH levels. The suspension prepared using 7 wt% DTPA/SW solution exhibited the most negative zeta potential value of −13.6 mV at pH = 12. In contrast, the suspension prepared using 1 wt% DTPA/SW solution resulted in the least negative zeta potential value of +6.83 mV at pH = 5.

The complexation process involves the coordination of metal ions with the lone pair electrons of the functional groups in DTPA, forming stable complexes. Nevertheless, at low pH values, DTPA molecules may undergo protonation, meaning they acquire an additional hydrogen ion (H+), resulting in a higher positive charge on DTPA molecules. This increased positive charge can limit the availability of negatively charged functional groups in DTPA for complexation with metal ions. The electrostatic repulsion between the positively charged DTPA molecules and the metal ions can hinder the formation of stable metal-DTPA complexes, leading to incomplete complexation. A high concentration of H+ ions can also compete with other ions, such as metal ions and counter ions from the colloidal particles, for available charged sites on the surface of colloidal particles. This increased competition can reduce the screening effect of ions, resulting in a decreased effective zeta potential of colloidal particles. At high pH values, On the other hand, the concentration of hydroxide ions (OH^−^) in the solution increases, resulting in a more alkaline environment. Hydroxide ions can screen or shield the surface charge of rocks, reducing the effective surface charge and leading to an increase in the negative magnitude of zeta potential. This is because the hydroxide ions can effectively neutralize or screen the positive charge on the rock surface, leaving a relatively higher negative charge, which contributes to the water-wetting behavior.

### 3.3 Sand pack flooding

**Fig. 14:**
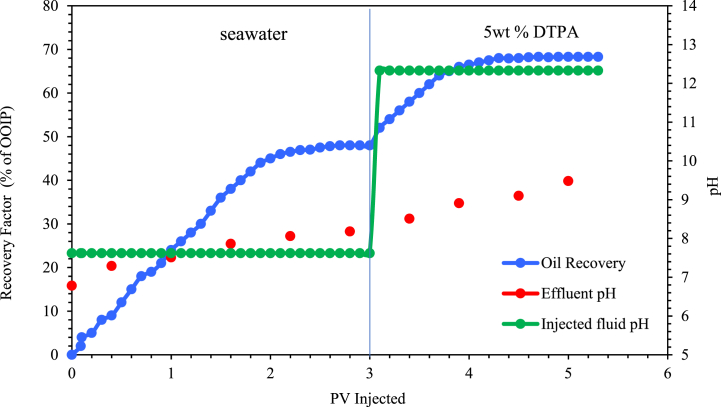
Oil recovery during SW and 5 wt% DTPA chelating agent flooding.

A sand pack flooding experiment was carried out using SW and 5 wt% DTPA solution. The sand pack was initially flooded with SW and then injected with the 5 wt% DTPA solution at pH 12.33. As shown in Fig. 14, injecting SW recovered 48 % of the original oil in place (OOIP), a value that increased to 68 % upon the injection of the 5 wt% DTPA solution. The increase in oil recovery can be attributed to the wettability alteration induced by the DTPA chelating agent injection. The pH of the flooding effluents was monitored over time and showed that the pH of the DTPA flooding effluent was lower than that of the injected fluid. This indicates that protons were lost from the injected fluid and adsorbed by the clays, resulting in alkaline conditions near the clay surfaces.

Rezaei Doust et al. (2011) considered these alkaline conditions as the primary cause of disrupting the system equilibrium, leading to the adsorption of crude oil components on clay surface [75]. Another factor could be the chelation and detachment of ${\text{Ca}}^{2+}$ ions from the rock surface, increasing its concentration in the effluent. Eq. (7) shows how the interaction between ${\text{Ca}}^{2+}$ and ${\text{OH}}^{-}$ can reduce the pH of effluent.

(7) $${\text{Ca}}^{2+}+{\text{OH}}^{-}={\left[\text{Ca}-\text{OH}\right]}^{-}$$

On the other hand, the injection of DTPA resulted in a gradual increase in the effluent pH over time. As illustrated in Fig. 14, after 5 wt% DTPA flooding, the effluent pH rose from 8.2 to 9.6. The increase in effluent pH is attributed to the high pH of DTPA and the removal of ions like ${\text{Ca}}^{2+}$ from both the solution and rock. Since the pH of the injected fluid was consistent, the rise in effluent pH over time can be due to the cation exchange that occurred between the rock and injected fluid. Additionally, there was a direct relation between the increase in the pH of flooding effluent and the improvement in oil recovery.

**Fig. 15:**
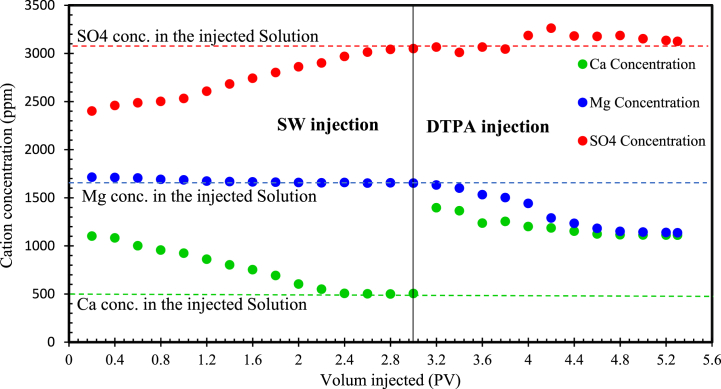
Concentration of different ions in sand pack flooding effluents.

Ion concentrations during both SW and 5 wt% DTPA flooding were determined using ICP analysis, and the results are illustrated in Fig. 15. As shown, during the initial stages of SW flooding, the concentration of ${\text{Ca}}^{2+}$ ion in the effluent was higher than its initial concentration in the injected fluid. This difference can be attributed to the substantial amount of ${\text{Ca}}^{2+}$ ions present in the connate water compared to SW (1300 and 499 ppm, respectively). It is worth noting that before the sand pack was flooded with SW, it originally contained 35 % connate water. Throughout the SW flooding process, this connate water was released, leading to a progressive increase in the ${\text{Ca}}^{2+}$ concentration in the SW effluent. However, by the end of the SW flooding phase, the ${\text{Ca}}^{2+}$ ion concentration had returned to its initial level. Furthermore, the ${\text{Ca}}^{2+}$ ion concentration in the DTPA effluent remained higher than its initial concentration throughout the entire DTPA injection process. This phenomenon is a result of the chelation of ${\text{Ca}}^{2+}$ ions by DTPA from both the rock and the solution, leading to an increase in their concentration in the flooding effluent. At the beginning of DTPA injections, the concentration of ${\text{Ca}}^{2+}$ ions in the effluents increased. Nevertheless, as time progressed, this concentration gradually decreased and eventually stabilized. This is because, at the first stages of DTPA flooding, there were more ${\text{Ca}}^{2+}$ ions for chelation, leading to rock dissolution and the leaching of these ions from both the rock and the solution. However, over time, due to the extensive chelation of these ions, the available ${\text{Ca}}^{2+}$ ions for chelation diminished, resulting in the observed reduction in their concentration in the flooding effluent.

On the other hand, the amount of ${\text{Mg}}^{2+}$ ions in connate water is not significantly higher than that in SW (1750 and 1641 ppm, respectively), resulting in a relatively consistent ${\text{Mg}}^{2+}$ ions concentration in the SW effluent. However, due to the multi-ion exchange (MIE) phenomenon, the concentration of ${\text{Mg}}^{2+}$ ions in the DTPA flooding effluents falls below its initial concentration (1136 vs. 1641 ppm). In fact, the chelation of metal ions such as ${\text{Ca}}^{2+}$ from the rock surface leads to the replacement of ${\text{Mg}}^{2+}$ ions on the rock surface. This, in turn, caused a decline in the concentration of ${\text{Mg}}^{2+}$ ions within the flooding effluent. Moreover, as demonstrated, the concentration of ${\text{Mg}}^{2+}$ in the effluent displayed a downward trend. This can also be attributed to the chelation of ${\text{Ca}}^{2+}$ ions, which results in the replacement of ${\text{Mg}}^{2+}$ ions, leading to a reduction in their concentration within the flooding effluent. The ${\text{SO}}_{4}^{2-}$ concentration in the SW was measured at 3069 ppm. Upon injecting the SW into the sand pack containing 35 % connate water, the process resulted in the precipitation of calcium sulfate. This was confirmed through effluent analysis, where the ${\text{SO}}_{4}^{2-}$ concentration initially decreased from 3069 to 2401 ppm and then began to rise again, indicating a reduction in calcium sulfate precipitation. During the injection of DTPA, the ${\text{SO}}_{4}^{2-}$ concentration in the effluent analysis increased and exceeded the sulfate concentration in the inlet solution, which was 3069 ppm. This suggests that DTPA effectively dissolved the calcium sulfate that had precipitated during the SW injection period. Additionally, injecting DTPA solution caused some ions (${\text{Al}}^{3+}$, ${\text{Cu}}^{3+}$, ${\text{Fe}}^{3+}$ and ${\text{Si}}^{4+}$) that were not initially present in the solution to be found in the flooding effluent. This can be attributed to the reaction of DTPA with the rock and the chelation of these ions from the rock surface. However, it should be noted that chelating agents should be injected in appropriate concentrations, as injecting them in inappropriate concentrations may cause damage to the reservoir [76].

## 4 Conclusions

This study aimed to investigate the effect of DTPA chelating agent on sandstone surface charge and oil recovery. For this purpose, wettability alteration, zeta potential, and sand pack flooding experiments were performed and the impact of different parameters on the DTPA performance were evaluated. The results that were obtained from this study are as follows:

• DTPA resulted in a more water-wet rock surface compared to LSW. The concentration of 5 wt% DTPA/SW changed the rock/oil contact angle from 143° to 23°, while LSW changed the rock/oil contact angle from 152° to 36°.
• DTPA at various concentrations was able to change the rock surface charge to a negative value. Moreover, increasing the pH and decreasing the salinity of the injected fluid led to an increase in the negative value of the zeta potential.
• The presence of crude oil in the solution made the zeta potential more negative by approximately 10–15 %.
• The performance of DTPA in changing rock surface charge was not affected by the presence or absence of PDIs in the solution, but tripling the concentration of these ions impaired its performance.
• Adding DTPA to LSW+1000 ppm Fe^3+^ solution caused a significant change in the zeta potential, which shifted from +6.51 mV to −14.73 mV.
• The flooding experiment showed that injecting 5 wt% DTPA chelating agent into the sand pack recovered 20.3 % of OOIP.
• The pH of DTPA flooding effluents increased over time, indicating rock dissolution and the reaction of DTPA with sandstone rock. Additionally, the concentration of ${\text{Ca}}^{2+}$ ions in the DTPA effluent increased, while the concentration of ${\text{Mg}}^{2+}$ ions decreased. This suggests that DTPA captured ${\text{Ca}}^{2+}$ ions from the rock, replacing them with ${\text{Mg}}^{2+}$ ions in the effluent.
• DTPA chelating agent dissolves rock and releases metal ions such as ${\text{Al}}^{3+}$, ${\text{Cu}}^{3+}$, ${\text{Si}}^{4+}$ and ${\text{Fe}}^{2+}$ that were not in the injected fluid.

## 5 Limitations

The usage of chelating agents for EOR presents potential benefits but is accompanied by challenges. Reservoir conditions such as temperature, pH, and mineral composition impact chelating agent performance, necessitating a comprehensive understanding of the reservoir for optimal dosing and injection strategies. Furthermore, the zeta potential instrument we employed cannot be used at reservoir conditions due to the following reasons: (1) they necessitate powdered samples, thereby not preserving the pore-space topology of the rocks, (2) they only account for the presence of the brine phase, and (3) they fail to capture the distribution of crude oil and brine within the reservoir pore space. Comparing zeta potential measurements on pulverized samples and intact rock samples highlights distinct differences in particle dispersion, surface area, and interparticle interactions. Pulverized samples, with finer particles, exhibit better dispersion and larger surface areas, enhancing zeta potential sensitivity. However, interparticle interactions may persist. Conversely, the natural structure of intact rock samples poses challenges for uniform dispersion and typically yields lower surface areas, potentially resulting in weaker zeta potential signals due to limited charge interaction opportunities. Addressing these challenges is crucial to ensure the successful application of chelating agents in sandstone reservoirs, thereby enhancing oil recovery while minimizing potential drawbacks.

## Data availability statement

Data included in article/supp. material/referenced in article.

## CRediT authorship contribution statement

**Mahsa Parhizgar Keradeh:** Conceptualization, Data curation, Formal analysis, Investigation, Methodology, Validation, Visualization, Writing – original draft, Writing – review & editing. **Seyyed Alireza Tabatabaei-Nezhad:** Methodology, Project administration, Resources, Supervision, Validation, Writing – review & editing.

## Declaration of competing interest

The authors declare that they have no known competing financial interests or personal relationships that could have appeared to influence the work reported in this paper.
